# Patterns of Chromosomal Variation, Homoeologous Exchange, and Their Relationship with Genomic Features in Early Generations of a Synthetic Rice Segmental Allotetraploid

**DOI:** 10.3390/ijms24076065

**Published:** 2023-03-23

**Authors:** Guo Li, Ying Wu, Yan Bai, Na Zhao, Yuhui Jiang, Ning Li, Xiuyun Lin, Bao Liu, Chunming Xu

**Affiliations:** 1 Key Laboratory of Molecular Epigenetics of Ministry of Education (MOE), Northeast Normal University, Changchun 130024, China; 2 Key Laboratory of Molecular Cytogenetics and Genetic Breeding of Heilongjiang Province, College of Life Science and Technology, Harbin Normal University, Harbin 150025, China; 3 Department of Agronomy, Jilin Agricultural University, Changchun 130118, China; 4 Jilin Academy of Agriculture, Changchun 130033, China

**Keywords:** polyploidization, chromosomal variation, homoeologous exchange, genomic feature, transposable elements, rice

## Abstract

Polyploidization is a driving force in plant evolution. Chromosomal variation often occurs at early generations following polyploid formation due to meiotic pairing irregularity that may compromise segregation fidelity and cause homoeologous exchange (HE). The trends of chromosomal variation and especially factors affecting HE remain to be fully deciphered. Here, by whole-genome resequencing, we performed nuanced analyses of patterns of chromosomal number variation and explored genomic features that affect HE in two early generations of a synthetic rice segmental allotetraploid. We found a wide occurrence of whole-chromosome aneuploidy and, to a lesser extent, also large segment gains/losses in both generations (S2 and S4) of the tetraploids. However, while the number of chromosome gains was similar between S2 and S4, that of losses in S4 was lower than in S2. HEs were abundant across all chromosomes in both generations and showed variable correlations with different genomic features at chromosomal and/or local scales. Contents of genes and transposable elements (TEs) were positively and negatively correlated with HE frequencies, respectively. By dissecting TEs into different classes, retrotransposons were found to be negatively correlated with HE frequency to a stronger extent than DNA transposons, whereas miniature terminal inverted elements (MITEs) showed a strong positive correlation. Local HE frequencies in the tetraploids and homologous recombination (HR) rates in diploids within 1 Mb sliding windows were significantly correlated with each other and showed similar overall distribution profiles. Nonetheless, non-concordant trends between HE and HR rates were found at distal regions in some chromosomes. At local scale, both shared and polymorphic retrotransposons between parents were negatively correlated with HE frequency; in contrast, both shared and polymorphic MITEs showed positive correlations with HE frequency. Our results shed new light on the patterns of chromosomal number variation and reveal genomic features influencing HE frequency in early generations following plant polyploidization.

## 1. Introduction

Polyploidization, or whole-genome duplication (WGD), has played an important role in the genome evolution of all higher plants [1,2,3,4]. Moreover, there is a long list of crops that are neo- or mesopolyploids (e.g., wheat, cotton, rape etc.), suggesting that polyploid plant species were favorable targets for domestication [5]. Newly formed or synthetic (artificially resynthesized) polyploid plants are generally associated with meiosis instability, generating aneuploidy and other types of chromosomal variations [6,7,8,9,10]. A major manifestation of unstable meiosis in polyploids is chromosome pairing irregularity, including multivalent and heteromorphic bivalent pairing (in allopolyploids, i.e., polyploid hybrids), which results in homeologous exchange (HEs) in allopolyploids and imprecise chromosome segregation in both auto- and allopolyploids [11,12,13]. Although the majority of these polyploidization-induced chromosome-level variants are likely deleterious and associated with fitness loss, some might be adaptive or meeting specific human requirements [14,15,16,17]. For example, many genome-sequenced neo- and meso-allopolyploid crops, such as those in *Brassica napus* [6,14,18,19,20,21], *Musa balbisiana* [22], *Fragaria ×*  *ananassa* [23], *Coffea arabica* [24], *Arachis hypogaea* [25], and *Triticum* [26] are found to contain HEs that underpin agriculturally important traits. Notwithstanding these important realizations and impressive progress, the trends of chromosomal variation and factors affecting HE immediately following polyploidization remain poorly understood.

It is well-established that a major deterministic factor influencing meiosis in both homoploid hybrids and allopolyploids is parental genetic divergence [11,27]. Moreover, it is generally believed that genetic polymorphism within genic regions (including protein-coding and noncoding regions) mainly plays such a role. However, genic regions make up only a small proportion of genome composition in most higher eukaryotes, while the bulk components of most plant genomes are transposable elements (TEs) and their derivatives [28,29]. Transposon insertion polymorphisms (TIPs) are also important sources of genomic variation [30,31]. For example, TIPs represent more than 50% of large insertion/deletions (indels) in the rice genome and account for ca. 14% of the genomic DNA sequence differences between the *japonica* and *indica* subspecies [30]. Furthermore, different TE classes map to different chromosomal regions and occupy spatially distinct “chromosome niches” [32]. For example, long-terminal repeat (LTR)-retrotransposons mainly colonize constitutive heterochromatic regions, such as centromeric and pericentromeric regions [33]. In contrast, most DNA transposons including miniature inverted-repeat TEs (MITEs) predominantly reside on chromosome arms and more distal telomeric regions that are mainly euchromatin [34,35]. In particular, MITEs are known to primarily reside within genic, noncoding regions such as upstream regulatory regions and introns [34,36]. Thus, it is perhaps not surprising that a strong negative association between TE contents and homologous meiotic recombination rates exists in diverse taxa [37,38,39,40,41]. However, whether similar trends also hold for homoeologous recombination in polyploids remains unknown.

We have reported that a synthetic segmental allotetraploid system between rice subspecies *japonica* and *indica* [42] was particularly suited to study the phenomena of chromosomal variation and HE at the initial stages of polyploidization. This is because meiotic pairing irregularity and homoeologous pairing widely occur in this set of newly formed polyploids [17,43], possibly due to the much lower extent of parental divergence than most allopolyploids parented by different species. However, many related issues, such as the trends of chromosomal variation and especially factors affecting HE occurrence, remain unexplored. In this study, we further studied these issues by performing whole-genome resequencing of two early generations (S2 and S4) of the synthetic rice segmental allotetraploids, with each generation containing 20 randomly chosen individuals. By using more in-depth analyses than the previous studies, we depicted genome-wide patterns of whole-chromosome gains and losses and revealed relationships between HE and various genomic features at both chromosomal and local scales.

## 2. Results

### 2.1. Patterns of Whole-Chromosome Gain and Loss in Early Generations of the Synthetic Rice Segmental Allotetraploids

**Figure 1:**
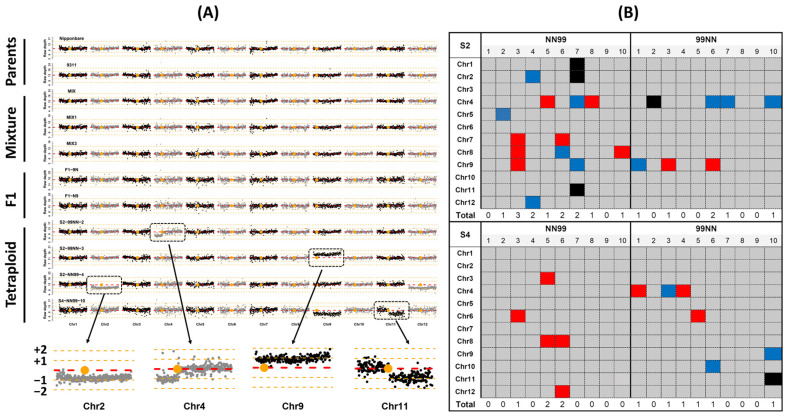
Representative karyotypes inferred from sequencing data and heatmap summary of aneuploidy in rice tetraploid S2 and S4 individuals. (**A**) Representative karyotypes. The genome-wide average sequencing depth is indicated by red dashed lines. The large orange dots represent centromeres. Each small black or grey dot represents a 1Mb window, and these two colors were used for distinguishing adjacent chromosomes. The *x*-axis shows the physical position of the window, and the *y*-axis shows the mean sequencing depth of the window. The four highlighted regions are four typical aneuploid chromosomes detected from four different tetraploid plants. The orange dashed lines from top to bottom are 1.5, 1.25, 0.75, and 0.5 of genome-wide depth, indicating +2, +1, −1, and −2 chromosomes in tetraploid. MIX, mixture of equal amount of Nippobare and 9311 data; MIX1, mixture of ¼ 9311 data and ¾ Nipponbare data; MIX3, mixture of ¾ 9311 data and ¼ Nipponbare data. Parents, their mixtures, and F1 individuals served as negative controls for aneuploidy identification. (**B**) Heatmap summary of aneuploidy. Every column stands for one individual and each row stands for a chromosome. Chromosome gain (red) and loss (blue) are shown in different colors. Large segmental gain and loss are shown in black color.

We reported that synthetic rice segmental allotetraploids constructed by inter-subspecific hybridization between *japonica* (cv. Nipponbare) and *indica* (cv. 9311) were meiotically unstable and generate whole-chromosome aneuploidies including both gain and loss of chromosomes [43]. However, because only one selfed generation (S4) was analyzed, it remained unclear if there existed differences in chromosomal variation between generations [43]. We first examined whole-chromosome gain and loss (i.e., aneuploidy) in two early generations of the rice synthetic segmental allotetraploids by using whole-genome resequencing. Several representative aneuploidy karyotypes are shown in Figure 1A. We sequenced 20 individuals (10 of each crossing direction) each of the S2 and S4 generations. We identified 13 (65%) and 9 (45%) aneuploid individuals in S2 and S4, respectively (Figure 1B). The reciprocals showed discernible differences in both generations, but the differences were not statistically significant (Fisher exact test *p*-value > 0.350). In S2, eight of the 10 individuals of rice tetraploids of Nipponbare (♀) × 9311 (♂) (NN99) showed either gain or loss (one individual showed both), while only five individuals in rice tetraploids of 9311 (♀) × Nipponbare (♂) (99NN) showed such changes; in S4, three individuals showed whole-chromosome gain (none showed loss) in NN99, while six individuals showed either gain or loss (none showed both) in 99NN (Figure 1B). Some individuals contained more than one aneuploid chromosome. Specifically, four and one plants of S2 had two and three aneuploid chromosomes, respectively, and two plants of S4 had two aneuploid chromosomes (Figure 1B; Table S1). Four (Chr1, Chr3, Chr6, and Chr10) and five (Chr1, Chr2, Chr5, Chr7, and Chr11) chromosomes showed neither gain nor loss in all individuals of S2 and S4, but only Chr1 was invariable across all individuals of both generations. In contrast, Chr4 showed the highest frequencies of gain/loss in both generations, being 30% and 15% in S2 and S4, respectively. The numbers of chromosome gains (nine events) vs. losses (ten events) were similar in S2; in contrast, more chromosome gains (eight events) than losses (three events) were found in S4 (Figure 1B, Figures S1 and S2). The numbers of chromosome gain between the two generations were similar (prop. test *p*-value = 1); however, the number of chromosome losses in S4 (three events) was obviously but not statistically lower than that in S2 (10 events) (prop. test *p*-value = 0.096). Together, these results suggest that the overall pattern of chromosome gain and loss are similar in earlier generations of the rice synthetic segmental allotetraploids irrespective of crossing directions. Moreover, while the frequency of chromosome gain is similar between the two generations, that of loss tends to decrease in the later generation.

Apart from whole-chromosome aneuploidies, we also noted large segmental gain and loss in some of the tetraploid individuals. Specifically, we found two plants in S2 (S2-99NN-2 and S2-NN99-7) had concomitant segmental gain in one chromosome (Chr.4) and loss in five other chromosomes (Chr1, Chr2, Chr5, Chr9, and Chr11). One individual in S4 also showed one segment loss (Figure 1B, Figures S1 and S2; Tables S1 and S2).

### 2.2. Relationships between Homoeologous Exchange and Genomic Features

Apart from whole-chromosome and large segmental gains and losses, it is known that homoeologous exchange (HE) occurs extensively in the synthetic rice segmental allotetraploids [17]. Here, we further studied HE and explored genomic features that likely impacted its occurrence and/or retention. We first identified all HEs in each individual of both S2 and S4 by using similar but more nuanced analyses than in our earlier study [17]. Briefly, we genotyped the chromosomal segments for all five possible homeologous ratios (9311: Nipponbare = 0:4, 1:3, 2:2, 3:1, or 4:0) based on homoeologous reads counts using a likelihood method (Materials and Methods) for each individual. The homoeologous recombination (crossover) regions between 9311 and Nipponbare segments in the tetraploids were determined based on the segmental ratios along each of the 24 different chromosomes.

**Figure 2:**
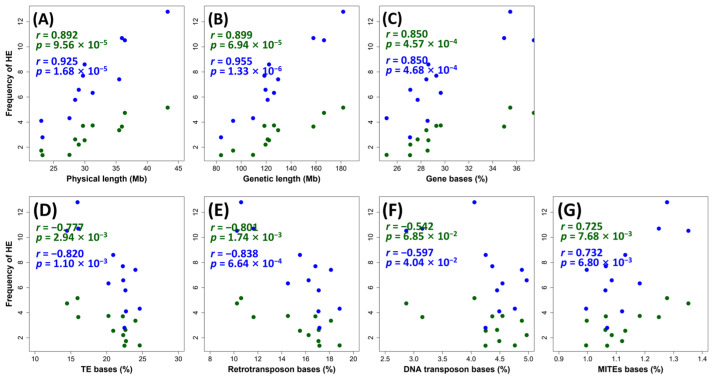
Correlation between HE frequency and different chromosome features. (**A**) physical length, (**B**) genetic length, (**C**) percentage of gene bases, (**D**) percentage of TE bases, (**E**) percentage of retrotransposon bases, (**F**) percentage of DNA transposon bases, (**G**) percentage of MITEs bases. Each dot represents a chromosome. The *x*-axis shows the genomic features of chromosomes. The *y*-axis shows the HE frequency of chromosomes. Chromosomes, correlation coefficients, and *p*-values of S2 and S4 plants are shown in green and blue colors, respectively.

Because whole-chromosome or large segmental gains/losses may obscure determination of HEs, we only used euploid plants (2n = 48, i.e., no whole-chromosome or segmental gain or loss) for further analysis. The average HE numbers per euploid individual were 33.16 and 89.45 in S2 (N = 6) and S4 (N = 11), respectively. The HE numbers on each chromosome varied in the ranges of 1.16 to 5.16 in the S2 plants and 2.91 to 12.73 in the S4 plants (Table S3). Chromosome 1 showed the highest HE numbers in both generations, while chromosome 12 and chromosome 10 showed the lowest HE numbers in S2 and S4, respectively (Table S3). We explored the possible correlations between the HE frequencies and various genomic features (Figure 2). We found that the HE number detected on each chromosome was positively correlated (*p*-values < 0.0001) with both of their physical and genetic lengths in both S2 and S4 plants (Figure 2A,B). For sequence features, HE number was positively correlated with the percent of aggregated total bases of genes (Figure 2C), whereas it was negatively correlated with the percent of aggregated total bases of transposable elements (TEs) (Figure 2D). We further separately analyzed the three major TE types, i.e., retrotransposons, DNA transposons and miniature terminal inverted elements (MITEs). We found that HE frequencies were significantly negatively correlated with the percent of retrotransposons (*p*-value < 1.74 × 10^−3^) in plants of both generations (Figure 2E). In contrast, HE frequencies were only weakly correlated with the percent of DNA transposons in plants of S4 (*p*-value = 0.04) and not correlated with the percent of DNA transposon in plants of S2 (*p*-value = 0.06) (Figure 2F). Interestingly, the percent of MITEs was significantly positively correlated with the numbers of HEs in plants of both S2 and S4 generations (Figure 2G). 

### 2.3. Relationship between Homoeologous Exchange and Homologous Recombination Rates at Local Scale

**Figure 3:**
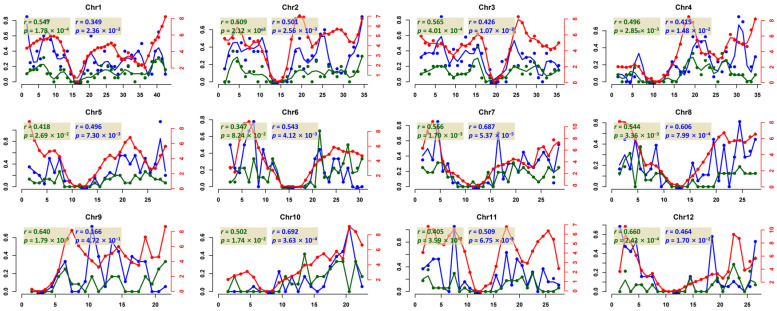
Distribution of local HE rates and local HR rate. Each dot stands for a 1Mb window. Local HE frequency (S2: green, S4: blue) and local HR rate (red) are shown in different colors. A smooth line was fitted using loess function (span = 0.2, degree = 2) for physical position with local HE frequency and HR rate, respectively. *X*-axis is the physical position. Y-axes are local HE rates (**left**) and local HR rates (**right**).

The foregoing results indicated that the correlation between HE frequency and genetic length was the highest among all tested chromosome features (Figure 2B). This may suggest a close relationship between the frequency of HEs (number per Mb) and homologous recombination (HR) rate (cM per Mb), given that genetic length is a direct manifestation of HR. This prompted us to further explore the relationship between HEs and HRs along each of the chromosomes. We first binned each chromosome into 1 Mb windows and calculated the local frequency of HEs in all tetraploid plants of both S2 and S4 (Materials and Methods). We then estimated the local HR rates (cM/Mb) using a publicly available HR dataset of the same inter-subspecific (japonica-indica) combination at the diploid level [44] for each window using a loess function method (Materials and Methods) as reported [45,46]. We found that the overall level correlation between local HEs and HR rates was highly significant (Pearson correlation test; *r* = 0.488, *p*-value < 2.2 × 10^−16^ in the S2 population and *r* = 0.483, *p*-value = < 2.2 × 10^−16^ in the S4 population). By partitioning the data into different chromosomes, we found that the local HE frequency and local HR rates showed highly concordant distribution profiles along each chromosome, i.e., lower rates of both HEs and HRs in centromeric/pericentromeric regions than arm and distal regions (Figure 3 and Figure S3). Significant correlations (*p*-value < 0.05) were detected in all chromosomes except for Chr6 in the S2 plants and Chr9 in the S4 plants (Figure 3), suggesting that additional factors may have exerted chromosome-specific effects. Interestingly, we found that local HE and HR rates showed non-concordant trends in some regions of some chromosomes in both S2 and S4 plants, such as the telomeric regions of the long arm of Chr1 and the short arm of Chr3 (Figure 3). To further validate these opposing trends, we used our previously published dataset that included 200 euploid S4 plants [17] to run the same analysis, and we found the same trends for these two chromosome regions (Figure S4).

### 2.4. Differential Contributions of Local Sequence Variants, TE Contents, and TE-Insertion Polymorphisms to HE Frequency

We further investigated impacts of variable aspects of parental genetic divergence on local HE frequency. First, for sequence variants, we calculated the numbers of single nucleotide polymorphisms (SNPs) and small (1–99 bp) and large (>100 bp) insertions/deletions (Indels) between 9311 and Nipponbare in each 1 Mb window and performed correlation tests between the frequencies of local sequence variants and HEs. We found that only the SNP frequency was significantly negatively correlated with HE frequency (*p*-value = 9.652 × 10^−5^ in S2 and *p*-value = 2.391 × 10^−4^ in S4). We further investigated the frequencies of TE-insertion polymorphisms (TIPs) and shared TEs between the parents in each 1 Mb window and evaluated their relationships with local HE frequency. Both the local frequency of TIPs and shared TEs were significantly negatively correlated with local HE frequency. Interestingly, the shared TEs showed higher correlation coefficients with HE frequencies at local scales (*r* = −0.300, *p*-value = 3.13 × 10^−9^ in S2 and *r* = −0.269, *p*-value = 1.24 × 10^−7^ in S4) than did TIPs (*r* = −0.167, *p*-value = 1.15 × 10^−3^ in S2 and *r* = −0.170, *p*-value = 9.94 × 10^−4^ in S4). By grouping shared TEs and TIPs into different classes, we found that frequencies of both shared retrotransposons and retrotransposon-TIPs were significantly negatively correlated with local HE frequency. Moreover, the shared retrotransposon frequency showed stronger correlation than retrotransposon TIPs (Table 1). The frequencies of both shared DNA transposons and DNA transposon-TIPs were not correlated with local HE frequency, except for the DNA transposon-TIPs in S4, for which the correlation was slightly significant (Table 1). Notably, both MITEs-TIPs and shared MITEs were significantly positively correlated with local HE frequencies except for shared MITEs in S4 (*p*-value = 0.059). Together, these results indicate that the local frequency of SNPs, TIPs, and shared TEs are all contributing factors to local HE occurrence but to variable extents; also, different classes of TIPs and shared TEs may show opposite relationships with local HE frequencies.

## 3. Discussion

Aneuploidy and other types of chromosomal variations have been reported in several newly formed or synthetic polyploid plants [6,7,8,9,10]. In this study, we observed similar numbers of chromosome gain and loss events in an earlier generation (9 gain and 10 loss events in 20 plants of S2) but more chromosome gain than loss events in a latter generation (8 gain and 3 loss events in 20 plants of S4). The results of S4 generation were in line with the observations in *Brassica napus* where the resynthesized allopolyploids showed a bias towards gain of extra chromosomes [6]. In our previous study, we found that 40% plants in a population of 312 S4 individuals of this synthetic rice segmental allotetraploid were aneuploidies [43]. Because all studied plants were from a single generation (S4), it remained unclear whether the aneuploid proportion may undergo changes with progression of generation. In this study, although the number of plants was small, we observed a very close proportion of aneuploid plants in the S4 population (45%), as in our earlier study [43], indicating that the smaller sample size is adequate for this purpose. Thus, our observation that a higher proportion of aneuploidies existed in the S2 generation (65%) suggests that with progression of generation, the aneuploid proportion tended to rapidly decline. This is important, because if the tetraploids are to be practically useful as direct cultivars, meiosis stability is essential. We also found there were more chromosome gains than losses in the S4 plants than in those of S2, suggesting that whole-chromosome losses are preferentially selected against. This is interesting, because in theory, meiotic irregularity would result in more losses than gains given that, while multivalents may result in equal probabilities of gains and losses, univalents (often co-occur with multivalents) almost certainly result in losses only due to univalent lagging in anaphase. Notably, however, our results are consistent with studies in other allopolyploid organisms [43,47,48].

HEs have been widely documented in different nascent natural or artificially synthesized plant polyploids [12,17,22,23,24,25,49,50]. In synthesized rice and wheat polyploids, HEs were found to exhibit biased distribution toward subtelomeric regions, suggesting that HEs are likely under the control of the same machinery as HRs [17,49]. However, in a recent study in allopolyploid wheat, rice, banana, peanut, *Brassica napus*, and *Arabidopsis suecica*, it was found that unlike HRs that preferentially take place in promoters and terminators of genes, HEs showed a strong bias for gene-body regions [49]. This suggests that there are different factors affecting the occurrence of HEs and HRs. In this study, we performed a more fine-scale comparison of local HE and HR rates and unveiled that their rates at local scale were generally correlated (Figure 3), which confirmed and extended the previous findings. Notwithstanding, we also observed local HE and HR rates showing non-concordant trends in some chromosomes or regions, suggesting that additional *cis*-factors may also be involved in the process of HE.

We have documented that homoeologous exchanges (HEs) occurred rampantly in progenies of this rice segmental allotetraploid, which generated wide-ranging phenotypic diversity [17]. However, how the variable genomic features and the inherent parental genetic divergence would impact the occurrence and/or retention of HEs have not been explored. Here, we show that the inherent genetic differences between the parental subspecies, including both contents and polymorphisms in genes and TEs, contribute to HE frequencies at either or both local and chromosomal scales. We find that the contents of genes and TEs are positively and negatively correlated with HE rates, respectively. This is as expected, given that genes should be more conserved (hence less divergent) than TEs between the parental subspecies. However, our finding that common or shared TEs played a greater negative role than TIPs in HE suppression is surprising. This suggests that sequence divergence between the parental common TEs is more relevant to homoeologous chromosome pairing than are large indels caused by presence/absence of TEs. Whether this has to do with their differential association with variable kinds of epigenetic modifications, and hence chromatin states [51], warrants further investigations. We also find that at local scale, in contrast to retrotransposons and DNA transposons that are either negatively correlated or did not show strong correlations with HE rates, MITEs show strong, positive correlations with HE rates. This may suggest that either MITEs per se play such a role due to their high sequence similarity (e.g., due to more recent mobilizations) between the parents, or that this occurs simply because of the preferential residence of MITEs at genic, noncoding regions [34,36]. Generalization of this phenomenon requires further studies in other plant taxa.

## 4. Materials and Methods

### 4.1. Comparative Genomic Analyses of Parental Genomes

The *japonica* cv. Nipponbare genome (MSU7.0) and the *indica* cv. 9311 genome (http://ricerc.sicau.edu.cn/RiceRC/download/downloadBefore, accessed on 19 October 2022) were downloaded and the chromosome sequences were aligned using nucmer with “-t 20 -l 50 -c 100 -maxmatch” parameters then filtered with “-m -I 90 -l 100” using delta-filter in MUMmer (version 4.0.0rc1) [52]. SNPs, structural variations (SVs), and copy number variations (CNVs) were identified using SyRI (version 1.4) with default parameters [53]. Small indels (<100 bp) and large indels (≥100 bp) were extracted from the SyRI output.

### 4.2. DNA Extraction, Sequencing, and Data Preprocessing

All plants, including Nipponbare, 9311, F1, and tetraploid plants were grown in a greenhouse under a 16/8-hour light/dark cycle and a 28/25 °C day/night temperature. Young leaves were harvested and frozen with liquid nitrogen. Samples were stored under −20 °C in a refrigerator. DNA were extracted using CTAB method. Whole-genome sequencing libraries were constructed using illumine TruSeq DNA sample preparation kit and sequenced on illumina HiSeq2000/HiSeq4000 platform according to the manufacturer’s instruction at BGI. All reads were filtered to remove low-quality reads and trimmed to 100 bp using Trimmomatic (version 0.39) [54] with parameter setting “ILLUMINACLIP: TruSeq3-PE-2.fa:2:30:10 LEADING:5 TRAILING:5 HEADCROP:10 CROP:110 MINLEN:75 TOPHRED33”.

### 4.3. Identification of SNPs in Syntenic Regions between Nipponbare and 9311

The short reads data of both Nipponbare and 9311 were mapped to the Nipponbare reference genome (version MSU7.0) using BWA mem (version 0.7.17) with “mem” and default settings [55]. The BAM files were processed using SortSam and MarkDuplicates; then, SNPs and indels were called and genotyped using HaplotypeCaller and GenotypeGVCFs in GATK (version 4.1.3.0) [56]. The SNPs were further filtered with “QD < 2.0 QUAL < 30.0 SOR > 3.0 FS > 60.0 MQ < 40.0 MQRankSum < −12.5 ReadPosRankSum < −8.0 ReadPosRankSum-8” using VariantFiltration tool in GATK. The remaining SNP sites were filtered to keep sites whose genotypes in Nipponbare were the reference genotype and in 9311 were the alternative genotype. The output of SyRI for comparative genomic analysis was processed to obtain the syntenic regions between Nipponbare and 9311 by excluding any non-sytenic regions, e.g., the SVs and CNVs as well as the up and down 100 bp regions adjacent to them. The overlapped SNPs identified by both short reads alignment and comparative genomic method in the syntenic regions between Nippobare and 9311 were selected for further analyses.

### 4.4. Allelic/Homeologous Specific Counting

Next, the clean data of reciprocal F1 hybrids and tetraploid samples were mapped against the rice Nipponbare reference genome (version MSU7.0) using BWA (version 0.7.17) with “mem” and default parameters [55]. The BAM files were sorted using Picard tools (version 2.18.27, http://broadinstitute.github.io/picard/, accessed on 19 October 2022). The number of allelic/homeologous specific reads were counted using ASEReadCounter tool in GATK (version 3.8) based on the SNPs in syntenic regions [57]. Sites with biased depth (two-fold) from either parent in any of the three control samples of N9-F1, 9N-F1, or parental MIX (mixture of equal amount parental data) were excluded from further analysis.

### 4.5. Chromosome Depth Painting and Identification of Chromosome Number Changes

Chromosomes were binned into 1 Mb windows. The mean depth of each window was calculated from counts of the allelic reads from both parents across all SNPs sites. Windows containing less than five SNP sites were removed. The windows were plotted along chromosomes by their position and depth. The cutoff of +1, +2, −1, −2 chromosome number changes were determined based on the 1.25×, 1.5×, 0.75×, and 0.5× of global depth. All samples were manually inspected to identify possible aneuploidy chromosomes and chromosome fragment gain or loss.

### 4.6. Detection of Homoeologous Exchanges (HEs)

To minimize the random sampling effect on the sequencing depth of few SNP sites, we sliced each chromosome into bins with 10 SNPs. Then, we calculated the reads count from either parents in each window and compared the ratio using a G-test of goodness of fit with varied null hypothesis of the parental ratio (9311:Nipponbare = 0:4, 1:3, 2:2, 3:1, or 4:0). The most like genotype with the lowest G value and highest *p*-value was assigned to each window. Continuous bins of the same genotype were merged into a fragment, and any fragment containing less than five bins was removed. The region between two fragments of different genotypes was classified as the homeologous recombination region.

### 4.7. Calculation of Percentages of Gene and TE Bases on Chromosomes

The chromosome features were calculated based on the Nipponbare reference genome (MSU7.0). Gene regions were extracted from the annotation file, and TE-related genes were excluded. The total bases covered by gene regions were summed and divided by the chromosome length for the percentage of gene bases for each chromosome. RepeatMasker (version 4.1.0) was used with the TIGR_Oryza_Repeats.v3.3 [58] as the custom library to annotated the repeats in the reference genome. The TE regions were grouped into retrotransposon, DNA transposon, and MITEs according to the annotations, and covered regions were summed for calculating the percentages of TE bases for every TE type on each chromosome.

### 4.8. Estimation of Local Recombination Rate

The rice high-density linkage map of 3267 markers was downloaded from https://rgp.dna.affrc.go.jp (accessed on 19 October 2022). The physical positions of markers on the rice genome (IRGSP build5) were downloaded from Oryzabase (https://shigen.nig.ac.jp/rice/oryzabase/, accessed on 19 October 2022) and then transferred into coordination on MSU7.0 using CrossMap (version 0.6.3) [59]. Markers were further curated to remove aberrant ones based on the distribution of physical positions on the MSU7.0 reference genome and genetic distances. The local recombination rate was estimated using the loess method in MareyMapOnline (https://lbbe-shiny.univ-lyon1.fr/MareyMapOnline/, accessed on 19 October 2022) [46].

### 4.9. Detection of TEs and TIPs between the Two Parents

The whole-genome resequencing data of the Nippobare and 9311 were used for TE detection using PoPoolation TE2 [60]. Briefly, the Nipponbare genome MSU7.0 and TE sequences of TIGR_Oryza_Repeats.v3.3 (http://rice.uga.edu/annotation_oryza.shtml, accessed on 19 October 2022) were used as reference genome and reference TEs. The reference genome was masked using RepeatMasker (version 4.1.0, http://repeatmasker.org/, accessed on 19 October 2022) with reference TE as library. Then, the merged reference of genome and TE sequences was created. The reads were mapped to the reference and further processed using PoPoolation TE2 in joint mode with default parameters. The detected frequency of the TE insertions of more than 0.95 or less than 0.05 were classified as the presence or absence of TEs in the genotype. TIPs were identified for TEs present in only one parental genotype. Common TEs were defined as TE insertions detected in both parents. All TEs were classified as retrotransposon, DNA transposon, or MITEs based on the annotation of TIGR_Oryza_Repeats.v3.3.

## Figures and Tables

**Table 1:** Correlation between local HE frequency and SNPs, indels, TIPs, and shared TEs.

	Pearson Correlation			
	S2		S4	
	*r*	*p*-Value	*r*	*p*-Value
SNPs	−0.200	9.65 × 10^−5^ **	−0.188	2.39 × 10^−4^ **
Small indels (1–99 bp)	−0.042	0.408	0.011	0.828
Large indels (>100 bp)	−0.057	0.269	−0.045	0.378
TIPs	−0.167	1.15 × 10^−3^ **	−0.170	9.94 × 10^−4^ **
Retrotransposon TIPs	−0.261	3.04 × 10^−7^ **	−0.238	3.27 × 10^−6^ **
DNA transposon TIPs	−0.073	0.158	−0.104	0.045 *
MITE TIPs	0.158	2.24 × 10^−3^ **	0.135	9.03 × 10^−3^ **
Shared TEs	−0.300	3.13 × 10^−9^ **	−0.269	1.24 × 10^−7^ **
Shared retrotransposons	−0.334	3.13 × 10^−11^ **	−0.313	5.82 × 10^−10^ **
Shared DNA transposons	−0.031	0.550	0.050	0.335
Shared MITEs	0.117	0.024 *	0.098	0.059

* *p*-value < 0.05, ** *p*-value < 0.01.

## Data Availability

The DNA sequencing data for this study have been submitted to NCBI SRA database and can be found under the following accession number PRJNA929285.

## Supplementary Materials

The following supporting information can be downloaded at https://www.mdpi.com/article/10.3390/ijms24076065/s1.
