# Supplementary material for: Patterns of Chromosomal Variation, Homoeologous Exchange, and Their Relationship with Genomic Features in Early Generations of a Synthetic Rice Segmental Allotetraploid
Source: Int J Mol Sci. 2023 Mar 23;24(7):6065. doi: 10.3390/ijms24076065 (PMC10094486; doi:10.3390/ijms24076065)

Figure S1. karyotype inferred from sequencing data for S2 samples. The genome-wide average sequencing depth was indicated by red dashed line. The large orange dots represent centromeres. Each small black or grey dot represents a 1Mb window and these two colors were used for distinguishing adjacent chromosomes. The x-axis shows the physical position of window and the y-axis shows the mean sequencing depth of the window. The orange dashed lines from top to bottom are 1.5, 1.25, 0.75 and 0.5 of genome-wide depth which indicate +2, +1, -1 and -2 chromosomes in tetraploid. MIX, mixture of equal amount of Nippobare and 9311 data; MIX1, mixture of ¼ 9311 data and ¾ Nipponbare data; MIX3, mixture of ¾ 9311 data and ¼ Nipponbare data. Parents, their mixtures and F1 individuals served as negative control for aneuploidy identification.

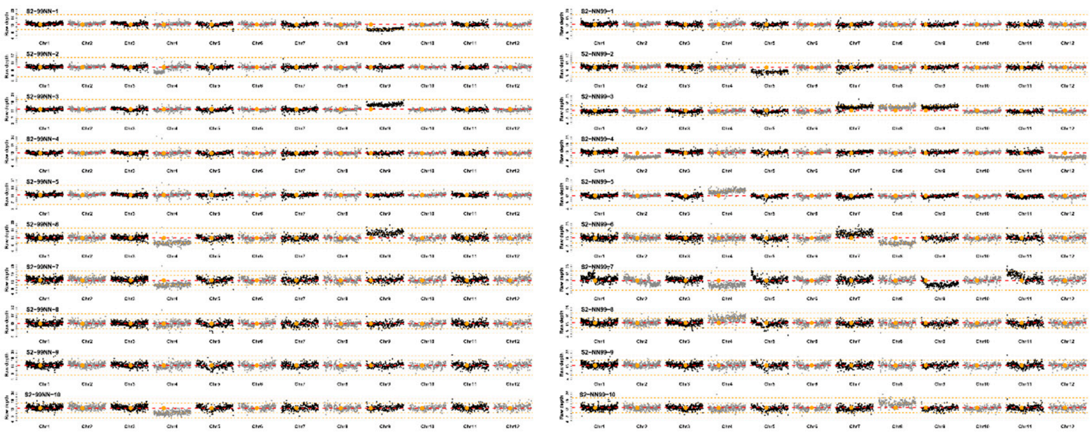

Figure S2. karyotype inferred from sequencing data for S4 samples. The genome-wide average sequencing depth was indicated by red dashed line. The large orange dots represent centromeres. Each small black or grey dot represents a 1Mb window and these two colors were used for distinguishing adjacent chromosomes. The x-axis shows the physical position of window and the y-axis shows the mean sequencing depth of the window. The orange dashed lines from top to bottom are 1.5, 1.25, 0.75 and 0.5 of genome-wide depth which indicate +2, +1, -1 and -2 chromosomes in tetraploid. MIX, mixture of equal amount of Nippobare and 9311 data; MIX1, mixture of ¼ 9311 data and ¾ Nipponbare data; MIX3, mixture of ¾ 9311 data and ¼ Nipponbare data. Parents, their mixtures and F1 individuals served as negative control for aneuploidy identification.

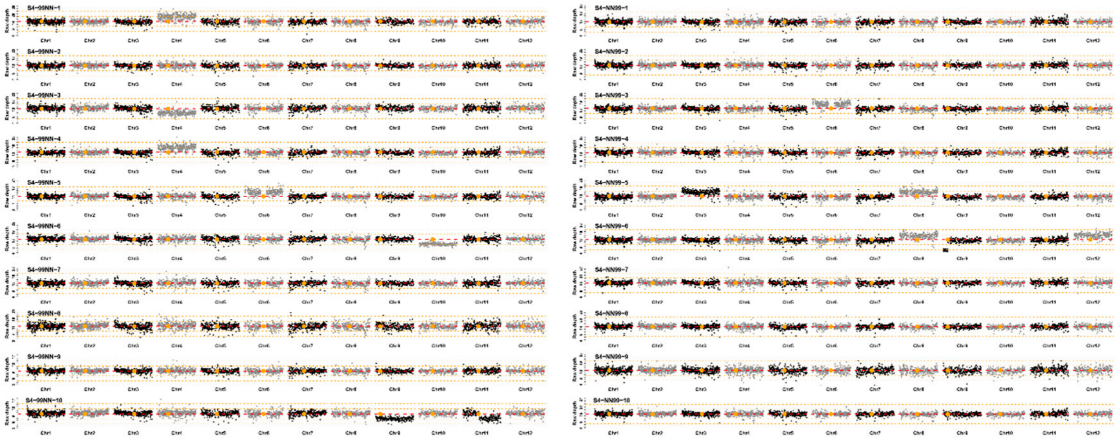

Figure S3. Distribution of local HE rates and local HR rate in S2 and S4 generation. Each dot stands for a 1Mb window. Local HE frequency (black) and local HR rate (red) are shown in different colors. A smooth line was fitted using loess function (span = 0.2, degree = 2) for physical position with local HE frequency and HR rate respectively. X-axis is the physical position. Y-axes are local HE rates (left) and local HR rates (right).

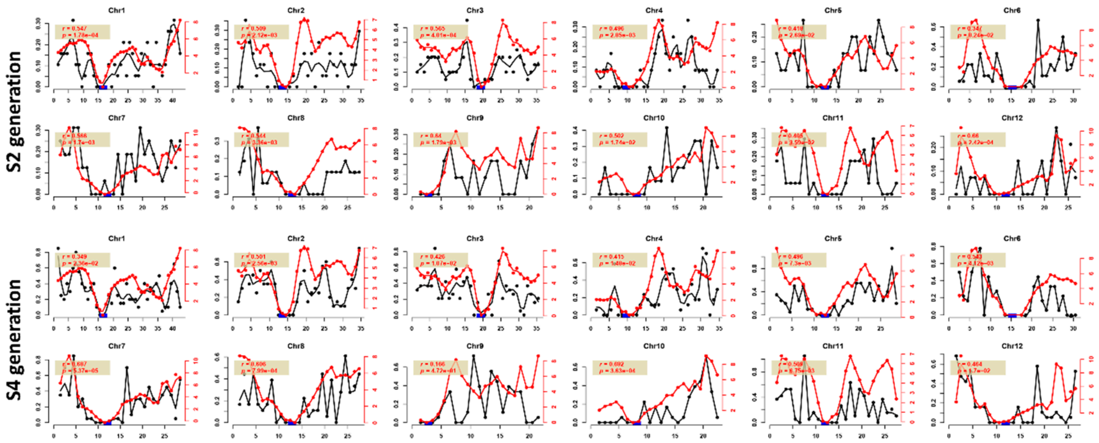

Figure S4. Distribution of local HE rates calculated from previously published dataset by Wu et al. 2021 and local HR rate. Each dot stands for a 1Mb window. Local HE frequency (black) and local HR rate (red) are shown in different colors. A smooth line was fitted using loess function (span = 0.2, degree = 2) for physical position with local HE frequency and HR rate respectively. X-axis is the physical position. Y-axes are local HE rates (left) and local HR rates (right).

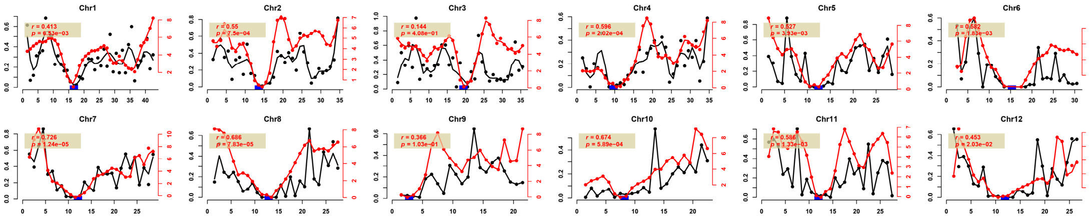

Supplement: Supplementary file 1 [file ijms-24-06065-s001.zip › Supplementary Figures.pdf]
