# Supplementary material for: Patterns of Chromosomal Variation, Homoeologous Exchange, and Their Relationship with Genomic Features in Early Generations of a Synthetic Rice Segmental Allotetraploid
Source: Int J Mol Sci. 2023 Mar 23;24(7):6065. doi: 10.3390/ijms24076065 (PMC10094486; doi:10.3390/ijms24076065)
Supplement: Supplementary file 1 [file ijms-24-06065-s001.zip › Supplementary Tables.pdf]

Table S1. Summary of karyotypes in S2 and S4 plants.

Karyotype	S2 Plant no.	S4 Plant no.	Total Plant no.
#1(-1,Chr4)	2	1	3
#2(+1,Chr4)	2	2	4
#3(-1,Chr5)	1		1
#4(+1,Chr6)		2	2
#5(+1,Chr8)	1		1
#6(-1,Chr9)	1	1	2
#7(+1,Chr9)	1		1
#8(-1,Chr10)		1	1
#9(-1,Chr4;+1,Chr9)	1		1
#10(-1,Chr2;-1,Chr12)	1		1
#11(+1,chr7,-1,Chr8)	1		1
#12(-1,Chr4;-1,Chr9)	1		1
#13(+1,Chr3;+1,Chr8)		1	1
#14(+1,Chr8;+1,Chr12)		1	1
#15(+1,Chr7;+1,Chr8;+1,Chr9)	1		1
#16(-1S,Chr04)	1		1
#17(-1LChr11)		1	1
#18(+1/2S,Chr01;-1L,Chr02;+2S,Chr11)	1		

Table S2. Number of plants with fragmental gain or loss in newly synthesized tetraploid rice.

	S2 generation		S4 generation	
chromosome	99NN	NN99	99NN	NN99
1	0	1	0	0
2	0	1	0	0
3	0	0	0	0
4	1	0	0	0
5	0	1	0	0
6	0	0	0	0
7	0	0	0	0
8	0	0	0	0
9	0	1	0	0
10	0	0	0	0
11	0	1	1	0
12	0	0	0	0
Total	1	5	1	0

Table S3. Number of HEs detected in euploid plants.

		Chr1	Chr2	Chr3	Chr4	Chr5	Chr6	Chr7	Chr8	Chr9	Chr10	Chr11	Chr12	Sum
S2	S2_9N_4	4	3	2	8	3	5	4	4	3	0	2	0	38
	S2_9N_5	6	1	6	2	1	2	4	3	2	3	2	2	34
	S2_9N_8	6	8	6	1	3	0	7	1	2	0	1	1	36
	S2_9N_9	4	4	6	1	5	3	5	4	2	0	2	0	36
	S2_N9_1	6	0	3	0	3	0	2	1	2	2	1	4	24
	S2_N9_9	5	2	3	4	1	6	1	1	2	3	3	0	31
	Mean	5.17	3.00	4.33	2.67	2.67	2.67	3.83	2.33	2.17	1.33	1.83	1.17	33.17
S4	S4_9N_2	12	12	7	3	3	4	11	6	4	3	12	4	81
	S4_9N_7	14	9	11	6	12	4	6	7	4	3	6	5	87
	S4_9N_8	9	13	9	3	8	3	8	6	6	7	10	3	85
	S4_9N_9	11	7	11	5	12	6	8	8	3	1	4	6	82
	S4_N9_1	11	8	14	6	13	10	5	9	7	1	6	5	95
	S4_N9_10	15	10	10	14	7	8	6	2	2	4	3	5	86
	S4_N9_2	16	8	14	3	11	9	5	8	7	0	5	3	89
	S4_N9_4	11	12	12	4	12	5	10	4	3	3	4	2	82
	S4_N9_7	13	16	15	11	12	6	13	7	0	3	7	3	106
	S4_N9_8	14	9	14	5	8	8	6	4	8	4	6	4	90
	S4_N9_9	14	18	13	12	13	8	9	1	2	3	5	3	101
	Mean	12.73	11.09	11.82	6.55	10.09	6.45	7.91	5.64	4.18	2.91	6.18	3.91	89.45
